# p38 MAPK and MDT-15 mediate SKN-1-dependent metformin-induced protection against mitis group streptococci in *Caenorhabditis elegans*

**DOI:** 10.1007/s11357-026-02405-6

**Published:** 2026-07-27

**Authors:** Ali Al Hatem, Boopathi Balasubramaniam, Alissa Villhauer, Ali K. Naji, Ransome van der Hoeven

**Affiliations:** 1 Department of Periodontics and Dental Hygiene, School of Dentistry, University of Texas Health Science Center, Houston, TX USA; 2 Iowa Institute for Oral Health Research, University of Iowa College of Dentistry, Iowa City, IA USA; 3 Department of Diagnostic and Biomedical Sciences, The University of Texas Health Science Center at Houston School of Dentistry, Houston, TX USA; 4 Department of Periodontics, University of Iowa College of Dentistry, Iowa City, IA USA

**Keywords:** *Caenorhabditis elegans*, Streptococcus, Metformin, SKN-1, MDT-15

## Abstract

Mitis group streptococci are opportunistic pathogens residing in the oral cavity, where they produce hydrogen peroxide (H_2_O_2_), a key virulence factor contributing to their pathogenesis. We previously demonstrated that these bacteria kill the nematode *Caenorhabditis elegans* through H_2_O_2_ production. Metformin, a widely used biguanide drug for managing blood glucose levels, has shown promising effects on aging by extending lifespan in worms and mice, and has also demonstrated potential cancer treatment. Additionally, pretreatment with metformin enhances the survival of worms and triggers an immune response via the p38 MAPK pathway against various pathogenic bacteria. Based on these observations, we investigated metformin's potential as a therapeutic agent against mitis group streptococcal infections and its underlying mechanisms in the current study. Pretreatment of worms with metformin provided significant dose-dependent protection from streptococcal-derived H_2_O_2_. This protective effect was attributed to the activation of the oxidative stress response transcription factor SKN-1/NRF2. Further analysis revealed that the p38 MAPK pathway and the transcription mediator complex protein MDT-15 are essential for the activation of SKN-1 by metformin. Interestingly, SKN-1 activity, mediated by MDT-15, was found to be independent of the p38 MAPK pathway. Our findings establish that metformin-mediated protection against mitis group streptococcal infections relies on the coordinated action of SKN-1, the p38 MAPK pathway, and MDT-15. These insights suggest that metformin could be a valuable therapeutic agent in combating these infections. Future research should focus on further elucidating the molecular mechanisms involved and exploring the potential clinical applications of metformin in treating these bacterial infections.

**Supplementary Information:**

The online version contains supplementary material available at https://doi.org/10.1007/s11357-026-02405-6.

## Introduction

Bacteria belonging to the mitis group streptococci are generally considered non-threatening inhabitants of the oropharynx [1]. However, these organisms can escape the oropharynx to cause a variety of invasive diseases such as infective endocarditis, orbital cellulitis, spondylodiskitis, community-acquired pneumonia, septic arthritis, and bacteremia in patients with neutropenia [2–10]. Moreover, pediatric cancer patients have shown to be more prone to bacteremia and subsequently develop viridians streptococcal shock syndrome [11, 12]. More importantly, unique strains of this group, *Streptococcus mitis* and *Streptococcus oralis* capable of causing bacteremia have been isolated from cancer patients that have undergone chemotherapy [5, 8, 13, 14]. These invasive strains are resistant to more antimicrobial agents than other viridians group streptococci species and consequently increase the risk of adverse patient outcomes [7, 15]. Members of this group have shown a propensity towards vancomycin tolerance and more recently some clinical isolates have shown the ability to rapidly develop a high level and durability of daptomycin resistance [16, 17]. Despite the clinical significances of these infections and the rising incidents of antibiotic resistance in this group of opportunistic pathogens, novel therapeutic strategies are required to combat these organisms.

Metformin which belongs to the biguanide class of drugs is widely used as an effective therapeutic to manage type II diabetes [18]. In addition to being used as a hypoglycemic medication, metformin has shown to decrease the growth of numerous cancer cells and is involved in the improvement of lifespan in model organisms [19, 20]. A few studies have also demonstrated metformin has antimicrobial properties for the treatment of pathogen infections [21–24]. Retrospective clinical studies have shown that patients with tuberculosis treated with metformin had less pulmonary cavities, less advancement of the disease and lower mortality rates compared to the non-metformin-treated group [24]. Pretreatment of hyperglycemic mice with metformin resulted in a significant reduction of airway *Pseudomonas* *aeruginosa and Staphylococcus aureus* bacterial loads [21, 22]. More recently, a study demonstrated the use of metformin to activate the innate immune response via the p38 mitogen-activated protein kinase (MAPK) pathway in the roundworm *Caenorhabditis elegans *[25]. The study highlights the potential use of metformin as a therapeutic against bacterial infections via the activation of the innate immune response. However, significant gaps in our knowledge exist to harness the potential of using metformin as therapeutic for the prevention and treatment of infections and the molecular mechanisms on how this drug exerts its effects.

The soil-dwelling nematode *C. elegans* is a well-established system to study evolutionarily conserved mechanisms of host defense and pathogen detection [26–28]. More importantly, this model is ideal to study pathophysiology in context of the whole organism. The worm lacks an adaptive immune system and professional immune cells to mount an immune defense. Instead, *C. elegans* relies on epithelial cells to protect against microbial infections. This provides an opportunity to discover the contribution of innate immune and stress responses in the absence of confounding effects of adaptive immunity and professional immune cells. In addition to being an excellent model for studying host–pathogen interactions, it is also an outstanding platform for drug discovery and elucidating mechanisms of drug action [29, 30].

Previous studies have shown metformin increases healthspan in *C. elegans* and non-diabetic mice [31–34]. More importantly, metformin-pretreatment extended the lifespan of worms compared to vehicle control treatment [20, 32]. These data suggest metformin may be a plausible pharmacological intervention to promote healthy human aging. The study further showed the conserved oxidative stress-responsive transcription factor, SKN-1/Nrf2 is essential for metformin healthspan benefits in the worm. SKN-1/NRF-2 has been shown to regulate phase II gene expression that provides protection against reactive oxygen species-induced oxidative stress damage in tissues that are associated with aging and many diseases [35–38].

MDT-15/MED15 has been identified as an evolutionarily conserved transcriptional mediator complex component in the regulation of fatty acid desaturation and the stress-response [39, 40]. Moreover, this mediator complex component has shown to be required for stress-response via the expression of *skn-1*-dependent genes. A study targeting amino acid catabolism identified multiple metabolic pathways as regulators of SKN-1 activity [39]. Remarkably, the activation of this *skn-1*-dependent response was shown to be mediated by MDT-15. Furthermore, MDT-15 has been shown to regulate oxidative metabolism in the presence of metformin [40]. More recently, MDT-15 in the worm has been identified in the xenobiotic-induced expression of PMK-1(p38)-dependent immune genes and protection against *P. aeruginosa *[41, 42].

Given that SKN-1 and MDT-15 have shown to regulate the oxidative stress response and are activated by metformin, we investigated the potential use of this therapeutic to enhance the innate immune response and confer protection against the mitis group streptococci. Our previous study showed that worms exposed to members of the mitis group survived significantly less and the killing was mediated by streptococcal-derived hydrogen peroxide (H_2_O_2_) [43]. In this study, we demonstrate that worms pretreated with metformin exhibit significantly increased survival compared to vehicle-treated controls when challenged with mitis group streptococci. This metformin-induced protection is mediated by SKN-1 via activation of the p38 MAPK pathway. Furthermore, we show that the transcriptional mediator complex component MDT-15 is essential for the mediated response. Our data suggests SKN-1, and MDT-15 mediates a protective response in the worms against the streptococcal-derived H_2_O_2_.

## Materials and methods

### Chemical reagents

Metformin was purchased from Sigma Chemical Co. (St. Louis, MO) and dissolved in sterile deionized water as a stock solution at a 1 M concentration and stored as aliquots at −20 °C.

### *C.elegans* and bacterial strains

*C. elegans* strains were cultured and maintained on Nematode Growth Medium (NGM) as previously described [44]. The strains used in this study are listed in supplementary Table S1, and some were outcrossed four times. Streptococcal strains were grown in Todd Hewitt broth supplemented with 0.3% yeast extract (THY), while *Escherichia coli* strains were cultured in Luria–Bertani (LB) broth. The bacterial strains used included *Streptococcus gordonii* DL1 Challis (wild type)*, Streptococcus mitis* VGS#3, *Streptococcus oralis* VGS#10, and *E. coli* OP50.

### Preparation of age synchronous population of worms

Gravid adult worms were collected from multiple NGM plates and treated with a bleach-sodium hydroxide solution to isolate eggs. Samples were centrifuged, and the resulting egg pellet was washed several times with M9 buffer before being incubated overnight in M9 to allow hatching. Age synchronized L1 larvae were then grown to the L4 stage on the appropriate media at 20 °C for subsequent assays.

### Preparation of metformin NGM and the growth of the worms

*E. coli* OP50 was spread on NGM plates supplemented with or without metformin (0, 1, 10, 50, 100 mM), vehicle control (sterile deionized water) and incubated overnight at 37 °C. Subsequently L1 worms were added to the OP50-lawn containing metformin or vehicle control plates. Worms were allowed to grow to the L4 stage at 20 °C to perform the various assays.

### Killing assays

For killing assays, bacterial strains were cultured in THY medium for 16 h, then plated on THY agar and incubated at 37 °C for 24 h in a candle jar. Subsequently, 6090 L4-stage larvae were moved to two replicate plates for each strain or condition and maintained at 25 °C under aerobic conditions [45]. Larval survival was assessed at multiple time points throughout the experiment, which was performed independently in triplicate.

### RNA interference

RNAi was induced by feeding L1-stage worms with *E. coli* HT115 expressing dsRNA against target genes from the L1 to L4 stages on NGM RNAi plates, with or without metformin supplementation. RNAi clones were sourced from the *C. elegans* RNAi library (Source Bioscience), and their identities were confirmed by sequencing. Clones absent in the library were generated as previously described [46].

### Amplex red assay

L1 larvae were cultured on NGM plates supplemented with or without 100 mM metformin at 20 °C until they reached the L4 larval stage. Worms were then washed off the plates using sterile M9 buffer and transferred to THY plates or NG plates seeded with overnight cultures of *S. gordonii* or *E. coli* OP50, respectively. After a 90-min exposure to the bacteria, worms were washed with reaction buffer. Thirty worms suspended in 50 µL of reaction buffer were dispensed into each well of a black, clear-bottom 96-well microplate. Subsequently, 50 µL of Amplex Red reagent (Invitrogen) was added to each well, and plates were incubated at room temperature in the dark for 60 min. Fluorescence was then measured using a SpectraMax iD3 plate reader with excitation at 540 nm and emission at 590 nm. A standard curve was generated using known concentrations of H₂O₂ (0.5, 1, 2, 4, 8, and 16 µM), prepared in triplicate and measured alongside the samples. H₂O₂ concentrations were calculated based on the standard curve, and the rate of H₂O₂ production was determined as previously described [47]. Three biological replicates were tested for each condition and the experiment was repeated three times.

### Quantification of intestinal bacteria

Worms pretreated with or without 100 mM metformin were exposed to *S. gordonii* on THY plates for 90 min. Following exposure, worms were washed in M9 buffer and anesthetized in M9 containing 25 mM levamisole for 30 min. Worms were then transferred to M9 supplemented with 25 mM levamisole, 1 mg/mL ampicillin, and 1 mg/mL gentamicin and incubated for 1 h to eliminate surface bacteria as previously described [48]. After antibiotic treatment, worms were washed twice again with M9 containing 25 mM levamisole. Groups of 10 worms were then mechanically disrupted in M9 containing 1% Triton X-100 using a motorized pestle. Serial dilutions of the worm lysates were plated on THY agar and incubated overnight at 37 °C, after which colony-forming units (CFUs) were counted. At least three replicates per condition were processed and the experiment was repeated three times.

### Disk diffusion assay

To determine whether metformin exhibits antimicrobial activity against *S. gordonii*, we performed a disk diffusion assay. Overnight cultures of *S. gordonii* were normalized, and spread evenly onto THY agar plates, which were then air-dried in a laminar flow hood for 10 min. Sterile paper disks were subsequently impregnated with varying concentrations of metformin (10, 50, and 100 mM) and placed onto the agar surface using sterile forceps. A 0.12% chlorhexidine gluconate solution was used as a positive control. Plates were incubated at 37 °C, and zones of inhibition were assessed at 24- and 48-h time points. The experiment was repeated three times.

### Fluorescence microscopy

To examine SKN-1 localization, transgenic worms expressing SKN-1B/C::GFP were grown on NGM or NGM RNAi plates, with or without metformin, until the L4 stage. Worms were then anesthetized with 0.1% sodium azide, mounted on 2% agarose pads, and imaged using a Nikon C2 Plus confocal microscope. The percentage of worms showing nuclear localization of SKN-1B/C::GFP in intestinal cells was scored as previously described [46]. Similarly, expression of the SKN-1-dependent gene *gst-4* was assessed by fluorescence microscopy in L4 transgenic worms carrying *gst-4p::*GFP, in the presence or absence of metformin. Imaging was performed using FITC and DAPI channels, and GFP expression levels were scored as previously described. All experiments were repeated at least three times.

### Protein lysate preparation and Western blot

Synchronized worms were transferred on both control (sterile water) and 100 mM metformin-seeded plates and incubated for 48 h or until they reach young adult stage. The worms were collected from each plate using M9 buffer and were washed twice with protein lysis buffer (50 mM Tris, pH 7.5, and 50 mM NaCl). Halt Protease and Phosphatase Inhibitor Single-Use Cocktail (Thermo Fisher Scientific) was added to each tube before lysis to a final concentration of 1X. The worms were then lysed on ice using a handheld homogenizer (Benchtop Lab Systems) for 2 min, followed by centrifugation at 10,000 rpm for 5 min at 4 °C. The resulting supernatants, containing protein lysates, were transferred to fresh vials. Protein concentration was determined using the Qubit 4 Fluorometer (Thermo Fisher Scientific) following the manufacturer’s protocol. For western blotting analysis, 20 µg of protein were loaded onto 5–15% gels (Bio-Rad Mini-PROTEAN® TGX™ Precast Gels) and transferred to a PVDF membrane using a turbo blotting kit (Bio-Rad Trans-Blot Turbo Transfer System, 0.2 µm PVDF Transfer Packs) according to the manufacturer’s instructions. The membrane was then blocked with EveryBlot Blocking Buffer (Bio-Rad) for 10 min at room temperature before incubation with primary antibodies at a 1:1000 dilution for 1 h with gentle shaking. After three washes with 1 × TBST (5 min each), secondary antibodies were added at a 1:2000 dilution and incubated for 1 h with gentle shaking at room temperature. The membrane was washed twice with 1 × TBST and once with 1 × TBS for 5 min respectively. Finally, the blots were developed using a HRP chemiluminescence substrate (SuperSignal™ West Pico PLUS Chemiluminescent Substrate), and imaged [49]. Primary antibodies used in this study: Phospho-p38 MAPK (Thr180/Tyr182) Antibody, Phospho-ASK1 (Thr845) Antibody, and α-Tubulin (DM1A) Mouse monoclonal antibodies. Secondary antibodies used: Anti-rabbit IgG, HRP-linked Antibody, and Anti-mouse IgG, HRP-linked Antibody.

### LC–MS/MS analysis

Proteins were extracted using 8 M urea made in 50 mM Tris pH 8.0 and quantified using Qubit 4 Fluorometer. Proteins from each sample underwent reduction, alkylation, and purification through chloroform/methanol extraction before digestion with sequencing-grade modified porcine trypsin (Promega). The resulting tryptic peptides were separated using a reverse-phase Ion-Opticks-TS analytical column (25 cm × 75 µm, 1.7 µm C18 resin) connected to an EASY-Spray nano-source, maintained at 55 °C with a Heater THOR Controller (Ion-Opticks). Peptides were initially trapped on a PepMap Neo trap column (300 µm × 5 mm) and subsequently eluted using a Vanquish Neo UHPLC nano system (Thermo Fisher Scientific), which kept samples at 11 °C before injection. Peptide elution occurred at a flow rate of 0.35 µL/min under a gradient starting at 98% Buffer A/2% Buffer B, shifting to 96/4 at 0.1 min, and gradually changing to 56/44 by 9.1 min. A column wash followed, transitioning from 45/55 at 11.7 min to 1/99 at 16.8 min, and finally re-equilibrating to 98/2 at 17.0 min. The eluted peptides were ionized by electrospray (2.5 kV) and analyzed with an Orbitrap Astral mass spectrometer (Thermo Fisher Scientific) [49]. Precursor spectra were recorded within a mass range of 380–980 Th at a resolution of 240,000, with an AGC target set to 200% and a maximum injection time of 3 ms (ms). DIA acquisition included 85 isolation windows (7 Th each) across the same mass range, with an AGC target of 100%, a maximum injection time of 3 ms, and an RF lens setting of 40%. Buffer compositions are as follows. Buffer A: 0.1% formic acid, 0.5% acetonitrile in water. Buffer B: 80% acetonitrile, 20% water, 0.1% formic acid.

### Post-processing of DIA analysis

Following data collection, LC–MS/MS spectra were analyzed using Spectronaut (Biognosys v19.1) against the UniProt *Caenorhabditis elegans* database (May 2024) with a direct DIA workflow. Protein and precursor identification thresholds were set at a 1% q-value cutoff, with decoy generation enabled. Protein inference was conducted using the IDPicker algorithm, with maxLFQ applied for quantification, MS2-level intensity measurements, no cross-run normalization, and protein grouping based on median peptide and precursor quantities. Protein MS2 intensity data were evaluated for quality with ProteiNorm [50], normalized using variance stabilization normalization [51], and statistically analyzed with proteoDA. Differential protein expression analysis was performed using linear models for microarray data (limma) combined with empirical Bayes (eBayes) smoothing [52]. Proteins with an FDR-adjusted *p*-value < 0.05 and a fold change greater than 1.5 were considered for bioinformatics analysis. The proteomics data have been submitted to the PRIDE consortium under the identifier PXD071991. Upregulated and downregulated proteins from each comparison were analyzed for their gene ontology using Metascape tool (v3.5.20250101) [53]. UniProt accessions for each protein were given as input and default settings were followed to retrieve the Gene Ontology (GO) terms including biological process and KEGG pathway. Heatmap was generated for significantly regulated proteins using the tool Heatmapper (http://www.heatmapper.ca/).

### Statistical analysis

All statistical analyses were performed using GraphPad Prism version 10.0 (GraphPad Software, San Diego, CA). Differences in the rate of H₂O₂ production were evaluated using one-way ANOVA, with pairwise comparisons assessed by unpaired *t*-tests with Welch’s correction. Differences in colonization levels were also evaluated using unpaired t-tests with Welch’s correction. Scored fluorescent micrographs were analyzed using chi-square and Fisher’s exact tests. For all analyses, *p*-values < 0.05 were considered statistically significant, and significant differences are indicated in the figures by asterisks next to the experimental conditions. Kaplan–Meier log-rank analysis was used to compare survival curves and calculate median survival, with *p*-values < 0.05 considered significant.

## Results

### Pretreatment with Metformin enhances the survival of the worms on mitis group streptococci

**Fig. 1:**
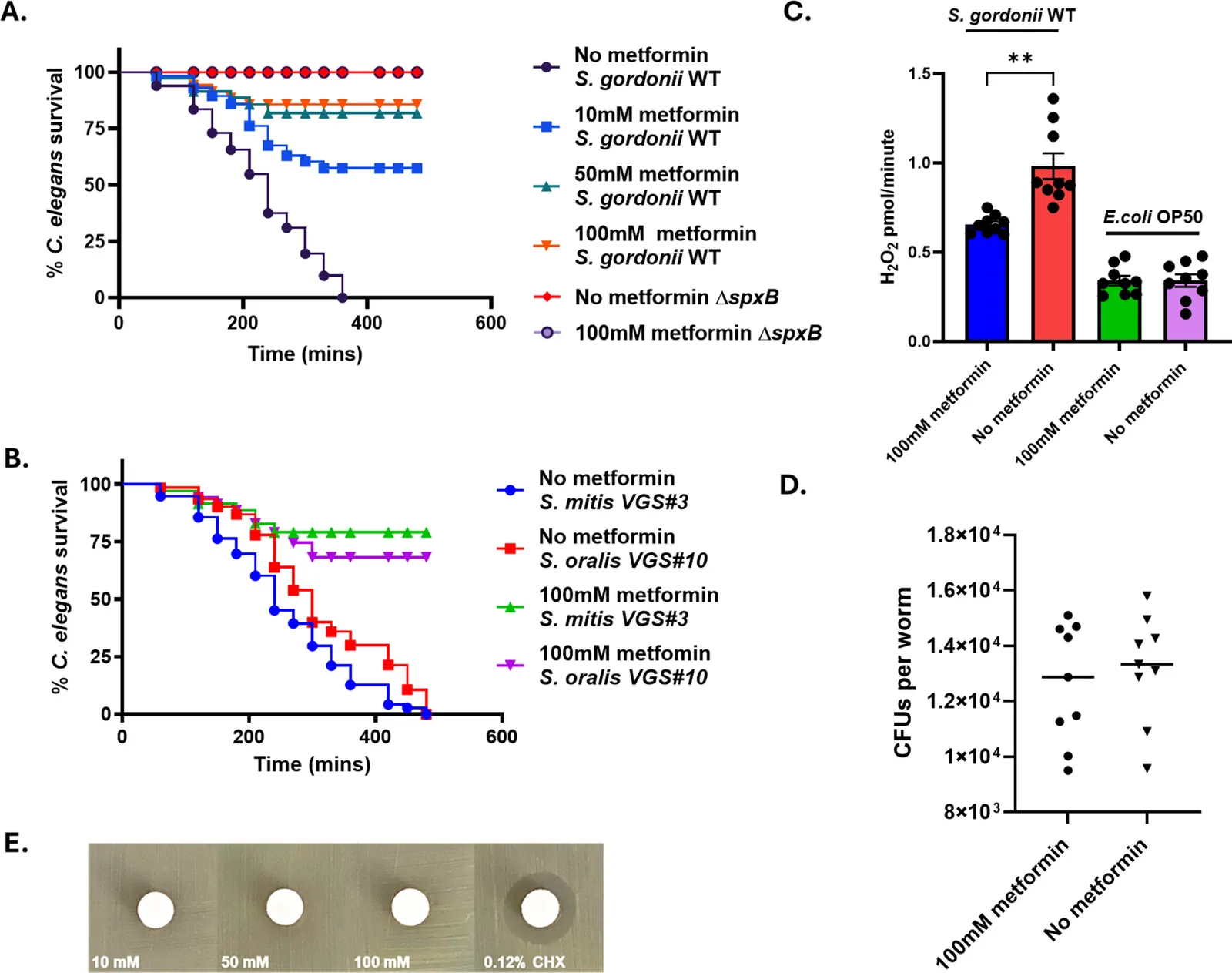
Metformin treated worms survived longer compared to vehicle control treated worms on mitis group streptococci. **A** Survival of N2 L4 larvae exposed to *S. gordonii* WT in the presence of 10 mM, 50 mM and 100 mM metformin and vehicle control (sterile water) on THY plates (****P < 0.0001). Survival of N2 L4 larvae was also observed on the *S. gordonii* Δ*spxB* mutant in the presence of 100 mM metformin and vehicle control on THY plates. **B** Survival of N2 L4 larvae exposed to *S. oralis* (VGS#3) and *S. mitis* (VGS#10)*,* in the presence of 100 mM metformin and vehicle control on THY plates (****P < 0.0001). Data represent experiments repeated at least three times, with 60–90 worms per condition. Survival curves were compared using Kaplan–Meier log-rank analysis, and median survival was calculated. *P*-values < 0.05 were considered statistically significant. **C** Amplex Red assay showing the rate of H₂O₂ production in L4 larvae pretreated with or without 100 mM metformin and exposed to *S. gordonii* WT (*P* = 0.0017) or *E. coli* OP50. Each experiment included three replicates and was repeated three times. **D** Colonization levels of *S. gordonii* WT were measured in N2 L4 larvae pretreated with or without metformin. For each condition, groups of 10 worms were homogenized and plated on THY agar, with a total of 90 worms analyzed across three independent experiments. **E** Representative images demonstrating the antimicrobial activity of metformin (10, 50, and 100 mM) against *S. gordonii* WT, evaluated using a disk diffusion assay, with 0.12% chlorhexidine gluconate as a positive control. The experiment was performed in triplicate

Using *S. gordonii* as a representative of the mitis group, we observed the survival of the worms increases in a dose-dependent manner on metformin compared to the vehicle control-treated worms (Fig. 1A). To extend these findings, we compared the survival of clinical strains of *S. mitis* and *S. oralis* as representative mitis group species and found that metformin pretreatment (100 mM) significantly increased worm survival compared to the untreated worms (Fig. 1B). Taken together, the data suggests that pretreatment with metformin enhances the survival of the worms when exposed to the mitis group streptococci. To explore the underlying mechanism, we used an Amplex Red assay to quantify H₂O₂ production in worms pretreated with or without metformin in the presence of *S. gordonii* or *E. coli*. We detected a modest but significant reduction in H₂O₂ levels in metformin-pretreated worms exposed to *S. gordonii*, whereas no differences were observed in worms maintained on *E. coli*, regardless of metformin treatment (Fig. 1C). These findings suggest that metformin may enhance survival by attenuating or scavenging H₂O₂ generated by *S. gordonii*. Given reports that metformin possesses antimicrobial and antibiofilm activity, we sought to determine whether the improved survival on *S. gordonii* was due to a direct effect on bacterial viability. We quantified CFUs of *S. gordonii* recovered from worms with or without metformin pretreatment and found no significant differences between conditions (Fig. 1D). Additionally, a disk diffusion assay revealed no zones of inhibition across all tested metformin concentrations, whereas a clear inhibitory zone was observed with 0.12% chlorhexidine gluconate (Fig. 1E). Together, these results indicate that the enhanced survival is not attributable to direct antimicrobial effects of metformin against *S. gordonii.*

### The oxidative stress response transcription factor SKN-1 mediates the metformin induced protection on mitis group streptococci

**Fig. 2:**
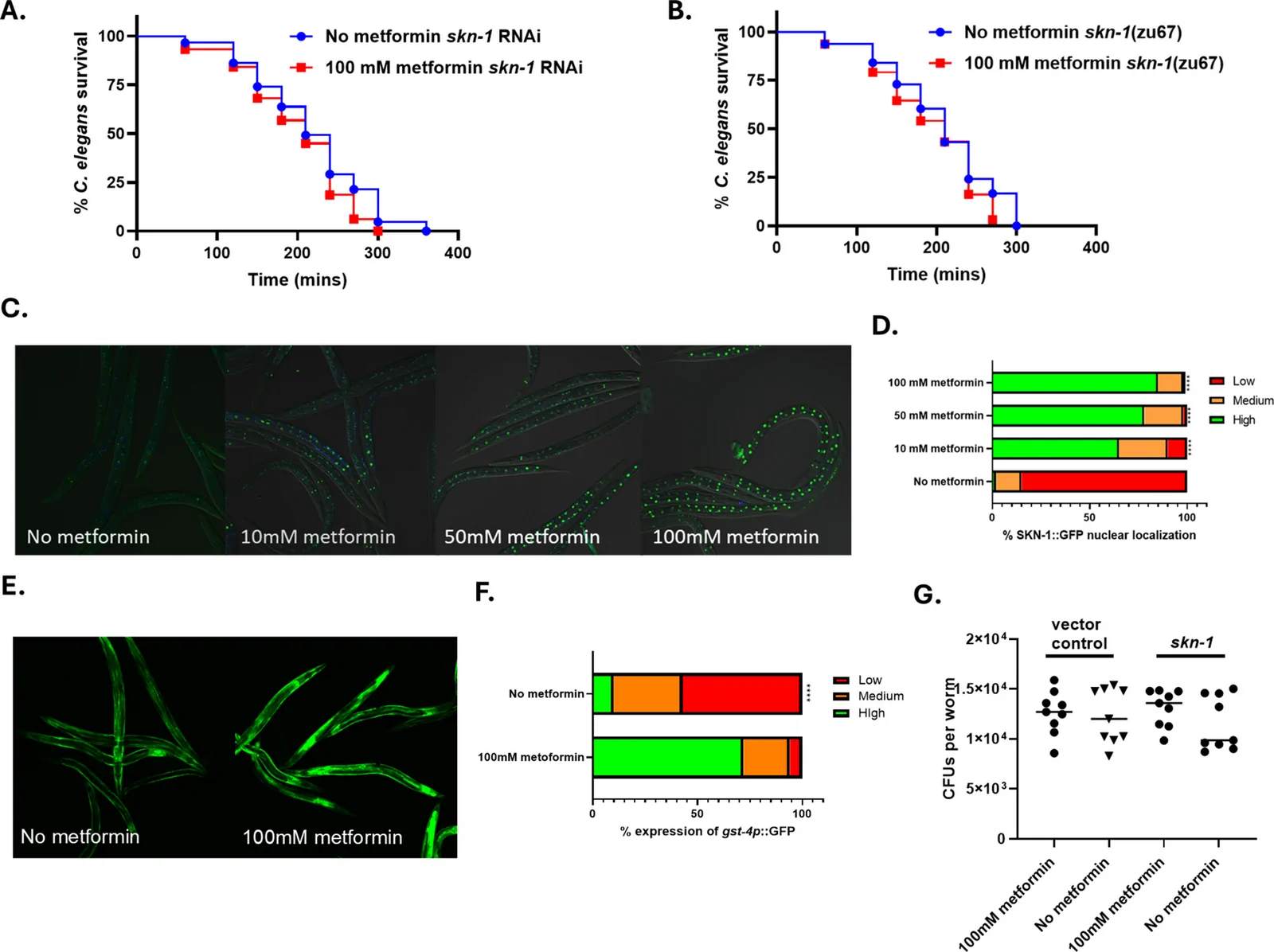
Enhanced survival of the worms on the pathogen in the presence of metformin is mediated via *skn-1*. **A** Survival of vector control treated, and *skn-1* knockdown worms exposed to *S. gordonii* WT in the presence 100 mM metformin and vehicle control (sterile water) on THY plates. **B** Survival of N2, and *skn-1*(zu67) mutant worms exposed to *S. gordonii* WT in the presence 100 mM metformin and vehicle control on THY plates. Data represent experiments repeated at least three times, with 60–90 worms per condition. Survival curves were analyzed using Kaplan–Meier log-rank tests, and median survival was calculated. *P*-values < 0.05 were considered statistically significant. **C **and** D** Representative images of the localization of SKN-1B/C::GFP in the presence of 10 mM, 50 mM, and 100 mM metformin and vehicle control and the degree of SKN-1B/C::GFP nuclear localization was scored, and the percentage of worms in each category is shown in the graph. **P < 0.0001; a total of more than 60 worms were analyzed, and the experiment was repeated three times. **E **and **F** The level of *gst-4*::GFP expression and the percentage of worms in each category, in the presence or absence of 100 mM metformin, are shown in the graph. More than 100 worms per condition were imaged, and the experiment was repeated three times. **P < 0.0001; a total of > 60 worms were analyzed for statistical comparison. **G** Colonization levels of *S. gordonii* WT were measured in *skn-1* knockdown and vector control treated L4 larvae pretreated with or without metformin. For each condition, groups of 10 worms were homogenized and plated on THY agar, with a total of 90 worms analyzed across three independent experiments

Given that metformin pretreatment reduced H₂O₂ levels in worms exposed to *S. gordonii*, we next investigated whether this protective effect involves activation of SKN-1, a key regulator of oxidative stress responses that has been shown to mediate metformin-induced increases in healthspan [20]. To test whether metformin-induced protection requires SKN-1, we knocked down *skn-1* expression and assessed worm survival on *S. gordonii* in the presence of metformin. No change in the survival was observed between the *skn-1* knockdown and vector control treated worms (Fig. 2A). Additionally, loss of *skn-*1 resulted in no significant difference in the survival of the worms on the pathogen in the presence or absence of metformin suggesting the enhanced survival on the pathogen in the presence of metformin was dependent on *skn-1* (Fig. 2B). Using a transgenic worm expressing SKN-1 fused to green fluorescent protein (GFP), we monitored the localization of SKN-1 in the intestinal cells of the worm in response to increasing concentrations of metformin (0, 10, 50, and 100 mM). Dose-dependent increase in the localization of SKN-1 was observed in the metformin treated worms, further confirming the activation of SKN-1 requires the biguanide drug (Fig. 2C and D). Furthermore, transgenic worms expressing *skn-1*-dependent gene *gst-4* fused to GFP were upregulated in response to treatment with metformin, validating our findings on SKN-1 (Fig. 2E and F). Lastly, we observed no significant difference in the colonization levels of *S. gordonii* WT between *skn-1* knockdown worms and vector control treated worms, in the presence or absence of 100 mM metformin (Fig. 2G). Together, these findings indicate that SKN-1 mediates metformin-induced protection of worms against *S. gordonii*.

### Metformin mediated SKN-1 activity requires the p38 MAPK pathway

**Fig. 3:**
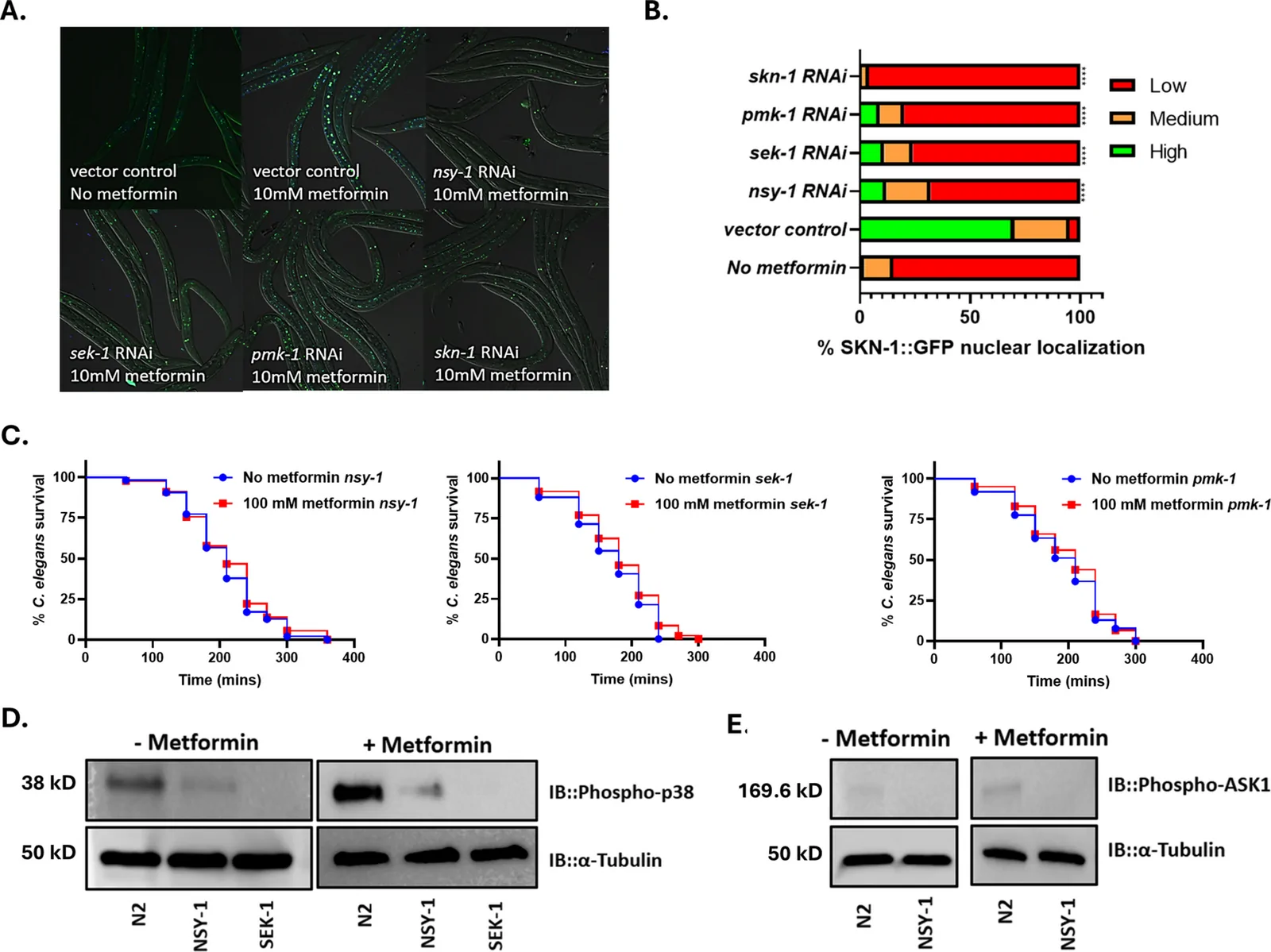
Metformin induced localization of SKN-1B/C::GFP is mediated by the p38 MAPK pathway. **A** Representative fluorescence images showing SKN-1B/C::GFP localization in worms subjected to RNAi knockdown of *nsy-1*, *sek-1*, and *pmk-1*, as well as vector control, in the presence of 10 mM metformin or vehicle control (sterile water). **B** The degree of SKN-1B/C::GFP nuclear localization was scored, and the percentage of worms in each category is shown in the graph. **P < 0.0001; more than 60 worms were analyzed per condition, and the experiment was repeated three times. **C** Survival of N2, *nsy-1*, *sek-1*, and *pmk-1* mutant worms exposed to *S. gordonii* wild type on THY plates, in the presence of 100 mM metformin or vehicle control. Data represent experiments repeated at least twice, with 60–90 worms per condition. Survival curves were analyzed using Kaplan–Meier log-rank tests, and median survival was calculated. *P*-values < 0.05 were considered statistically significant. **D** Western blot analysis of PMK-1 phosphorylation in N2, *nsy-1*, and *sek-1* mutant worms in the presence or absence of 100 mM metformin. Phosphorylation of PMK-1 was partially reduced in *nsy-1* mutants, while phospho-p38 was completely absent in *sek-1* mutants. α-Tubulin was used as a loading control. The experiment was repeated three times. **E** Western blot was used to analyze the level of phosphorylation of NSY-1 (ASK-1) in N2 worms in the presence or absence of 100 mM metformin. Phosphorylation of NSY-1 was increased in the presence of 100 mM metformin. α-tubulin was used as a loading control. The experiment was repeated 3 times

We and others have shown the p38 MAPK pathway that activates SKN-1 in response to numerous stress conditions [43, 46, 54, 55]. The p38 cascade is comprised of the MAPKKK, NSY-1 (ASK-1 homologue), the MAPKK, SEK-1 and MAPK PMK-1. To determine if metformin activates SKN-1 via the p38 MAPK signal axis, we knocked down *nsy-1*, *sek-1* and *pmk-1* in SKN-1B/C::GFP metformin-treated worms and observed the localization of SKN-1 relative to the vector control-treated worms. A significant decrease in the levels of localization was observed in *nsy-1*, *sek-1*, and *pmk-1* knockdown worms compared to the vector control-treated worms (Fig. 3A and B). As previously reported, we compared the survival of *nsy-1*, *sek-1*, and *pmk-1* mutant worms in the presence and absence of metformin. No significant change in the survival of the worms was observed between the metformin treated and untreated mutant worms (Fig. 3C). To confirm if PMK-1 is activated by metformin and requires NSY-1 and SEK-1, we performed Western blot analysis to determine the levels of phosphorylation of PMK-1 in the presence and absence of metformin. The levels of phosphorylation of PMK-1 were significantly higher in worms pretreated with metformin relative to the untreated worms. Loss of NSY-1 and SEK-1 significantly reduced the levels of phosphorylation of PMK-1 in the metformin treated samples (Fig. 3D). Furthermore, we observed that phosphorylation of NSY-1 was increased in the presence of metformin compared to WT control and *nsy-1* mutant, suggesting the p38 MAPK pathway mediates the activation of SKN-1 via metformin to provide protection against the mitis group streptococci (Fig. 3E).

### Peroxiredoxins and IRE-1 do not mediate the activation of SKN-1 via p38 MAPK

**Fig. 4:**
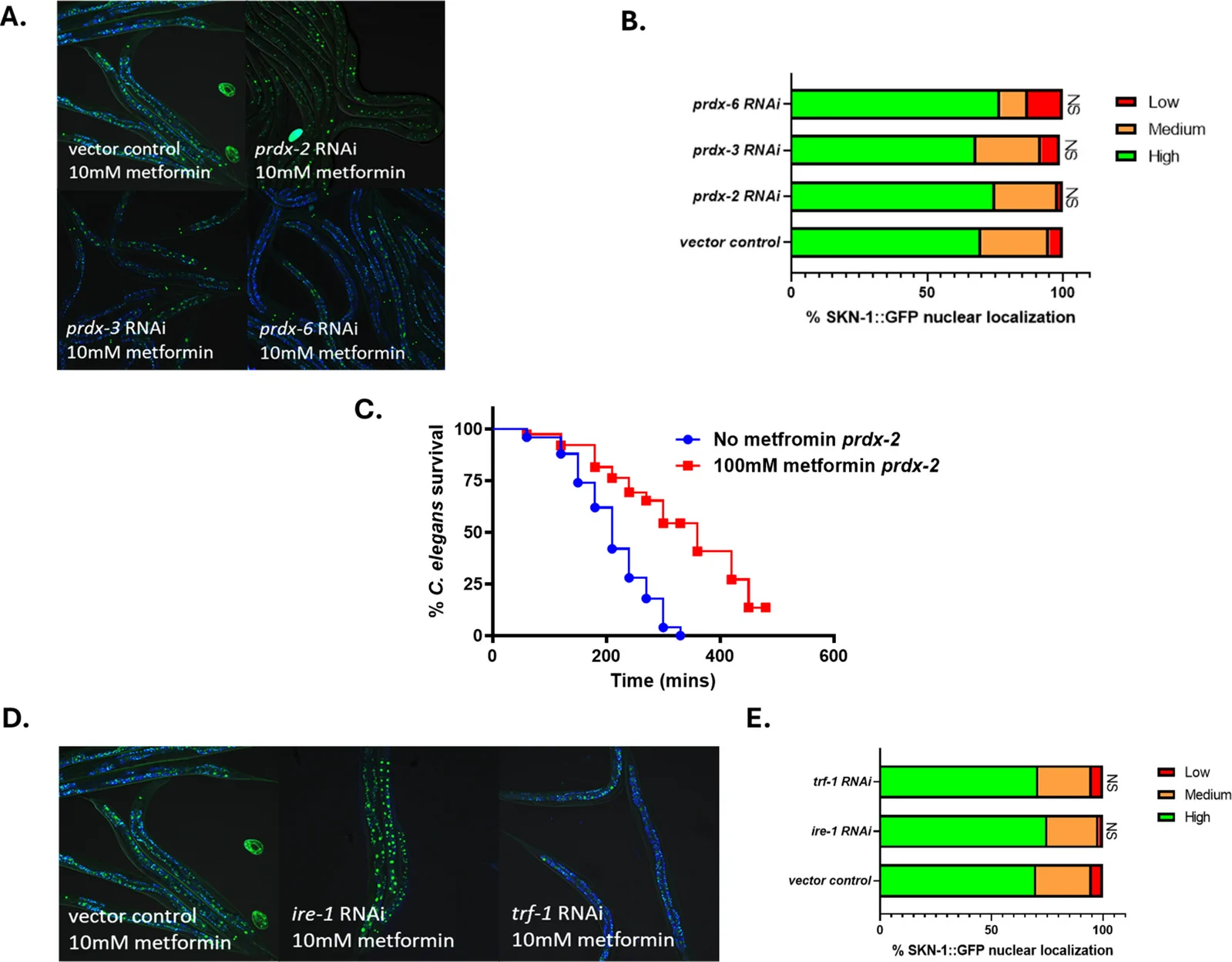
Peroxiredoxin PRDX-2 and IRE-1 do not mediate the metformin induced activation of SKN-1. **A** Representative fluorescence images showing SKN-1B/C::GFP localization in worms subjected to RNAi knockdown of *prdx-2*, *prdx-3*, and *prdx-6*, as well as vector control, under conditions of 100 mM metformin or vehicle control. **B** The degree of SKN-1B/C::GFP nuclear localization was scored, and the percentage of worms in each category is shown in the graph. **P < 0.0001; more than 60 worms were analyzed per condition, and the experiment was repeated three times. **C** Survival of N2, and *prdx-2* mutant worms exposed to *S. gordonii* WT in the presence 100 mM metformin and vehicle control (sterile water) on THY plates. Data represent experiments repeated at least three times, with 60–90 worms per condition. Survival curves were analyzed using Kaplan–Meier log-rank tests, and median survival was calculated. *P*-values < 0.05 were considered statistically significant. **D** Representative fluorescence images showing SKN-1B/C::GFP localization in worms subjected to RNAi knockdown of *ire-1* and *trf-1* knockdowns and vector control in the presence of 10 mM metformin and vehicle control. **E** The degree of SKN-1B/C::GFP nuclear localization was scored, and the percentage of worms in each category is shown in the graph. **P < 0.0001; more than 60 worms were analyzed per condition, and the experiment was repeated three times

Peroxiredoxins function as chaperones and redox sensors. A previous study reported the activation of PMK-1 by metformin was mediated by the peroxiredoxin PRDX-2 [32]. More importantly, they speculated SKN-1 was activated by p38 through PRDX-2 and regulated the metformin enhanced longevity in the worm. To verify if the metformin treatment activates SKN-1 via PRDX-2, we investigated the localization of SKN-1B/C::GFP in *prdx-2* knockdown worms relative to the vector control treated worms. We also knocked down two other peroxiredoxins, namely *prdx-3* and *prdx-6* in the presence of metformin and observed the localization of SKN-1B/C::GFP in the intestinal cells of the worm. We observed greater localization of SKN-1 in *prdx-2* knockdown worms, while no significant change was detected in the *prdx-3* and *prdx-6* knockdowns (Fig. 4A and B). The data implies that PRDX-2, PRDX-3, and PRDX-6 are not required for metformin activation of SKN-1. Importantly, knockdown of *prdx-2* resulted in the activation of SKN-1 in the absence of metformin suggesting a role as chaperone and not an activator of the p38 MAPK (data not shown). While loss of *prdx-2* resulted in significant decrease in the survival of the worms in the presence of metformin further confirming *prdx-2* is not required for metformin mediated protection in the worms (Fig. 4C). Furthermore, we observed that the *prdx-2* mutant worms were more susceptible to *S. gordonii* compared to WT worms in the absence of metformin (data not shown). The data suggests PRDX-2 is important for the detoxification of streptococcal-derived H_2_O_2_ but does not facilitate the metformin mediated protection against mitis group streptococci.

The endoplasmic reticular stress sensor, IRE-1 and the ortholog of human TRAF4, TRF-1 have shown to regulate the activation of SKN-1 through the p38 MAPK pathway [56]. We investigated if the knockdown of *ire-1* and *trf-1* influences the localization of SKN-1 in worms in the presence of metformin. We observed no significant change in the levels of localization of SKN-1 between the *ire-1* and *trf-1* knockdown worms in the presence of metformin relative to the vector control treated worms (Fig. 4D and E). Taken together, we did not identify a sensor or upstream components responsible for metformin-mediated activation of SKN-1 via the p38 MAPK pathway, suggesting that metformin activates SKN-1 through a mechanism independent of ER stress-associated or classical ROS-induced pathways.

### MDT-15 influences the activation of SKN-1 and metformin mediated protection in the worms

**Fig. 5:**
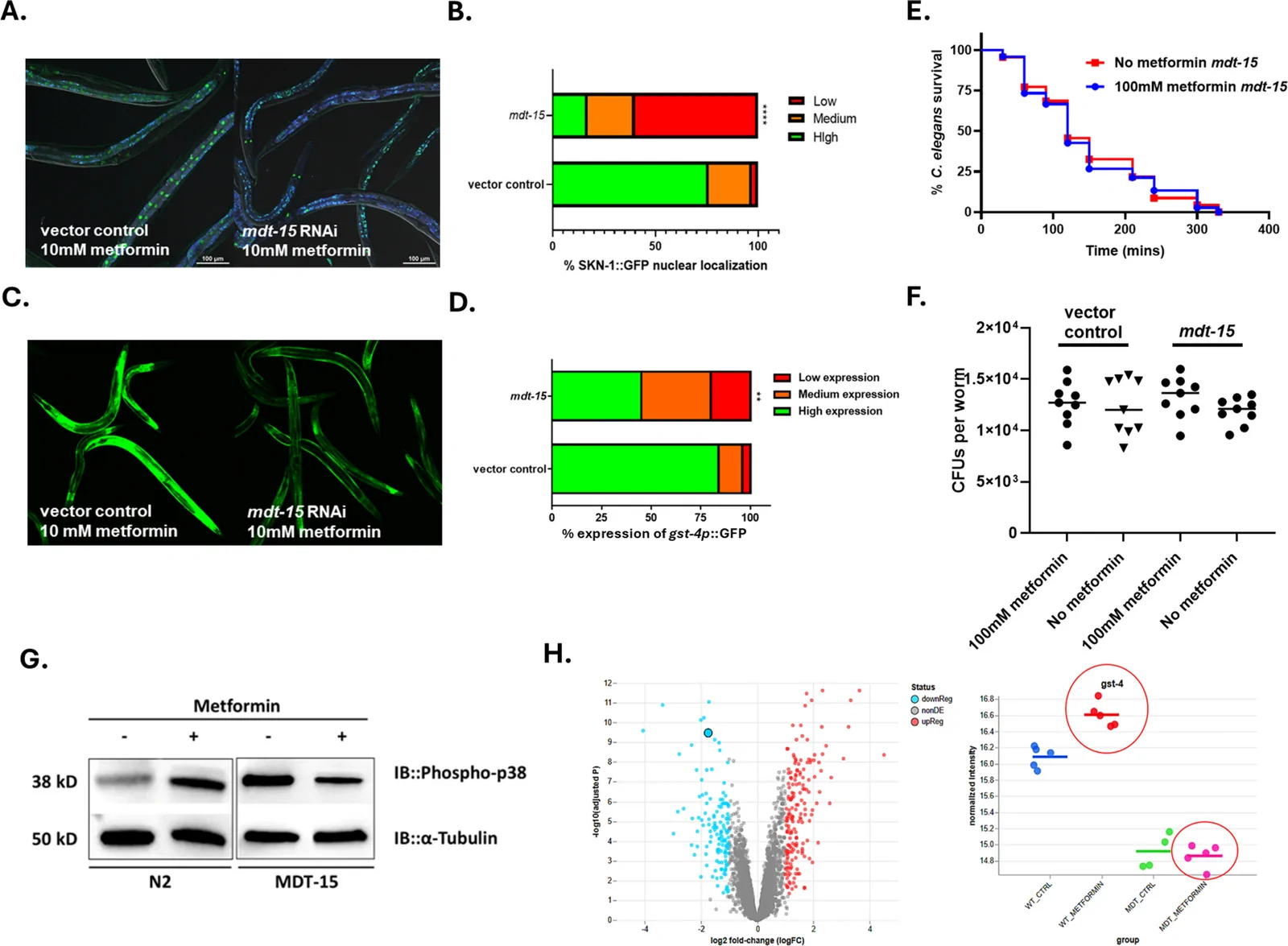
Metformin induced *skn-1*-dependent protection is mediated by MDT-15. **A** Representative fluorescence images showing SKN-1B/C::GFP localization in worms subjected to RNAi knockdown of *mdt-15* and vector control, in the presence or absence of 10 mM metformin and vehicle control. **B** The degree of SKN-1B/C::GFP nuclear localization was scored, and the percentage of worms in each category is shown in the graph. **P < 0.0001; more than 60 worms were analyzed per condition, and the experiment was repeated three times (**C** and **D**). The level of *gst-4*::GFP expression and the percentage of worms in each category fed on *mdt-15* RNAi or vector control, in the presence or absence of 10 mM metformin, are shown in the graph. More than 100 worms per condition were imaged, and the experiment was repeated three times. **E** Survival of N2 and *mdt-15* mutant worms exposed to *S. gordonii* WT in the presence 100 mM metformin and vehicle control (sterile water) on THY plates. Data represent experiments repeated at least three times, with 60–90 worms per condition. Survival curves were analyzed using Kaplan–Meier log-rank tests, and median survival was calculated. *P*-values < 0.05 were considered statistically significant. **F** Colonization levels of *S. gordonii* WT were measured in *mdt-15* knockdown and vector control treated L4 larvae pretreated with or without metformin. For each condition, groups of 10 worms were homogenized and plated on THY agar, with a total of 90 worms analyzed across three independent experiments. **G** Western blot analysis of PMK-1 phosphorylation in *mdt-15* mutant and N2 worms in the presence or absence of 100 mM metformin. Loss of *mdt-15* did not significantly affect PMK-1 phosphorylation. α-Tubulin was used as a loading control. The experiment was repeated three times. **H** Volcano plot represents the expression patterns of proteins in *mdt-15* mutant worms relative to N2 worms in the presence of 100 mM metformin. Relative normalized intensity of GST-4 in N2 and *mdt-15* mutant worms when exposed to metformin

We first observed the localization of SKN-1B/C::GFP in *mdt-15* knockdown and vector control treated worms to determine if MDT-15 influence the activation of SKN-1 in response to metformin. A significant decrease in the localization of SKN-1 was observed in *mdt-15* knockdown compared to vector control treated worms in the presence of metformin (Fig. 5A and B). Similarly, expression of *gst-4* fused to GFP was significantly reduced in *mdt-15* knockdown worms when compared to vector controls, consistently supporting our findings on the role of MDT-15 in SKN-1 activation (Fig. 5C and D). More importantly, there was no significant difference in the survival of *mdt-15* mutant worms on *S. gordonii* in the presence and absence of metformin suggesting the involvement of MDT-15 (Fig. 5E)*.* No significant difference was observed for colonization levels of *S. gordonii* WT in *mdt-15* knockdown and vector control treated worms in the presence or absence of 100 mM metformin (Fig. 5 F). Taken together, these data suggest that MDT-15 regulates the activity of SKN-1 and the protection of worms against *S. gordonii* in the presence of metformin. Additionally, we determined the levels of phosphorylation of PMK-1 in the absence of MDT-15. We measured the levels of phosphorylation of PMK-1 in the *mdt-15* mutant worms in the presence and absence of metformin. A significant increase in the levels of phosphorylation of PMK-1 was observed in *mdt-15* mutant worms compared to the WT **(**Fig. 5G). Additionally, loss of MDT-15 does not significantly affect the phosphorylation levels of p38 upon metformin treatment. Even though the levels of phosphorylation of PMK-1 increased in the absence of *mdt-15* compared to WT, we did not observe an increase in the levels of SKN-1 localization suggesting *mdt-15* is required for the regulation of the activity of SKN-1 through a direct or indirect interaction when treated with metformin. We next determine the protein profiles of WT and *mdt-15* mutant worms in the presence or absence of metformin using LC–MS/MS analysis (Supplementary Table 2). We observed an upregulation of phase II genes in response to oxidative stress in worms, which was dependent on *mdt-15* in the presence of metformin, as evidenced by GST-4 levels (Fig. 5H). Proteomic and GO analyses collectively indicate that MDT-15 mediates both basal physiological homeostasis and adaptive responses to metformin in *C. elegans* (Fig. S1A and B). Under normal conditions, MDT-15 mutant worms exhibited enrichment of stress- and defense-associated processes, including responses to external stimuli, endocytosis, amino acid catabolism, and endoplasmic reticulum (ER) unfolded protein response signaling (Supplementary Tables 2 and 3). Concurrently, KEGG pathway analysis suggested that pathways associated with TGF-β signaling, Wnt signaling, ubiquitin-mediated proteolysis, branched-chain amino acid degradation, and cytochrome P450–mediated drug metabolism were downregulated in the *mdt-15* mutant (Supplementary Table 4). These alterations, together with proteomic signatures of increased defense responses and ER stress, suggest that loss of MDT-15 leads to broad metabolic imbalance and constitutive activation of cellular stress pathways under basal conditions. In response to metformin, *mdt-15* mutants showed differential enrichment of pathways involved in fatty acid metabolism, unsaturated fatty acid biosynthesis, ER protein processing, and cofactor metabolism compared with treated WT worms, whereas pathways associated with pyruvate metabolism, fatty acid degradation, ATP-dependent chromatin remodeling, and cytochrome P450 activity were downregulated. (Supplementary Table 4). This shift is indicative of a compensatory metabolic adaptation favoring lipid remodeling and altered energy utilization. Consistent with these observations, proteomic data further suggest that MDT-15 regulates multiple biological processes including metabolic pathways, chromatin organization, and stress-response programs to mediate a protective response to metformin. Together, these findings support a model in which MDT-15 is essential both for maintaining physiological homeostasis and for enabling metabolic flexibility and adaptive stress responses upon metformin exposure, in agreement with its established role as a central regulator of stress adaptation and metabolic remodeling in *C. elegans* [57, 58].

## Discussion

In this study, we demonstrated that metformin pretreatment in *C. elegans* provides protection against the mitis group streptococci. We showed that metformin-induced protection against these opportunistic pathogens is mediated by the oxidative stress response transcription factor SKN-1, and its activation is mediated by the p38 MAPK pathway. Additionally, we showed that MDT-15 is required for the metformin-induced expression of *skn-1*-depdendent genes independent of the p38 MAPK. Furthermore, Proteomic analysis indicates that MDT-15 mediates metformin-induced protection in *C. elegans* by regulating processes such as metabolism, chromatin organization, and cellular functions. Taken together, our data suggest that metformin protects the worms from streptococcal-derived H_2_O_2_ by activating an oxidative stress response via p38-SKN-1 signaling axis and MDT-15.

Our findings further strengthen the evidence that metformin exerts protective effects beyond its conventional role in glycemic control. In a murine model of acute pneumonia and sepsis, metformin treatment significantly reduced infection-associated morbidity and suppressed inflammatory responses during *Streptococcus pneumoniae* (SPN) infection compared to untreated controls [59]. These results suggest that metformin modulates host–pathogen interactions by limiting excessive inflammation, a major contributor to tissue damage and poor outcomes in severe pneumococcal disease. Notably, metformin pre-treatment prior to SPN challenge appeared to prevent hyperinflammation, indicating potential prophylactic benefits for high-risk populations. Supporting this observation, proteomic analysis in worms revealed that metformin downregulates immune activation while enhancing oxidative stress defenses mediated by SKN-1. This dual mechanism attenuation of pro-inflammatory signaling coupled with activation of cytoprotective antioxidant pathways likely underpins the observed reduction in disease severity.

A previous study had shown that metformin enhances innate immunity in *C. elegans* by activating the PMK‑1/p38 MAPK pathway, providing broad protection against both Gram-negative and Gram-positive pathogens [25]. Our findings reveal that metformin’s protective effects extend beyond p38 MAPK signaling, implicating the oxidative stress regulator SKN‑1 in defense against mitis group streptococci. SKN‑1, a key transcription factor downstream of p38 MAPK, governs oxidative stress and detoxification pathways in *C. elegans*. This SKN‑1 dependency parallels observations in HepG2 cells expressing an Nrf2-responsive Antioxidant Response Element (ARE) luciferase reporter, where reporter activity increased with rising metformin concentrations [60]. Consistent with these findings, metformin treatment elevated antioxidant and stress response proteins in murine livers, reduced hepatic oxidative damage, and leading to increased lifespan and healthspan in mice.

An important unresolved question concerns the mechanism by which metformin activates the p38 MAPK pathway to initiate SKN-1 mediated protection against mitis group streptococci. Evidence from previous research indicates that PRDX-2 mediates p38 MAPK activation, contributing to lifespan extension in *C. elegans *[32]. However, our findings indicate that PRDX-2 is critical for worm survival in response to streptococcal-derived H₂O₂ but does not influence SKN-1 localization in the presence of metformin. We also examined the role of IRE-1, which has been implicated in p38 activation under oxidative stress [56]. SKN-1 localization was significantly higher in *ire-1* mutants treated with metformin, suggesting that SKN-1–mediated protection occurs independently of IRE-1. Collectively, these observations point to alternative mechanisms and pathways that regulate metformin-induced activation of SKN-1, which further warrants investigation.

Numerous studies have shown that AAK-2, an α-isoform of AMP-activated protein kinase (AMPK) in *C. elegans*, is activated in response to metformin and functions as a key metabolic regulator of stress responses and lifespan maintenance [20, 40, 61]. In addition, AAK-2 has been implicated in controlling both the localization and activity of SKN-1, linking its role to longevity. Recent evidence indicates that components of the p38 MAPK pathway specifically *pmk-1*, *tir-1*, *nsy-1*, and *sek-1* are upregulated during *Bacillus thuringiensis* infection and are regulated by AAK-2 [62]. These findings suggest that AMPK is not only an evolutionarily conserved energy sensor but also a critical mediator of host–pathogen interactions. Therefore, it is plausible that metformin influences SKN-1 activity through AAK-2 by modulating p38 MAPK signaling. Future studies will clarify the role of AAK-2 in modulating p38 MAPK activity in response to metformin.

Beyond SKN‑1, we identified MDT‑15 as a critical cofactor required for SKN‑1 activation following metformin treatment. MDT‑15, a subunit of the conserved Mediator complex, is critical for oxidative stress responses and SKN‑1 target gene expression [39]. It physically interacts with SKN‑1 and enables its transcriptional program under oxidative stress. Our data extend these findings by showing that MDT‑15 knockdown abolishes metformin-induced SKN‑1 activity and downstream protection against streptococcal-derived H₂O₂. Notably, the SKN‑1–MDT‑15 interaction occurs independently of the p38 MAPK pathway, highlighting a distinct regulatory axis and an opportunity to explore Mediator complex activation in response to metformin. This is particularly intriguing considering prior work showing that the xenobiotic toxin RPW‑24 requires MDT‑15 to induce both PMK‑1-dependent immune effectors and PMK‑1-independent detoxification genes [42]. These studies further suggest that MDT‑15 can function either downstream of or in parallel to the p38 MAPK pathway, underscoring its context-dependent regulatory roles. Emerging evidence indicates that MDT‑15, together with Box C/D snoRNPs, regulates the Ethanol and Stress Response (ESRE) mitochondrial surveillance through fatty acid metabolic programs, linking lipid homeostasis to innate immunity and host survival under pathogenic conditions [63]. Consistent with a role in metabolic regulation, analysis of our proteomic data revealed that the MDT‑15–dependent fatty acid desaturases FAT‑6 and FAT‑7 are downregulated in response to metformin treatment. This finding suggests a shift in lipid metabolism and raises the possibility of an altered ESRE-driven stress response. Further studies will be required to define how these metabolic changes contribute to mitochondrial stress signaling and host defense. In this context, MDT‑15 is known to interact with metabolic regulators such as SBP‑1 and NHR‑49 under metformin exposure [40]. To investigate SBP‑1’s role, we examined SKN‑1 localization in the presence of metformin and observed significantly increased nuclear accumulation (data not shown). Moreover, SBP‑1 knockdown further enhanced SKN‑1 nuclear localization, suggesting that SBP‑1 functions as a negative regulator of SKN‑1 activity and does not mediate SKN-1 mediated protection against streptococcal derived H_2_O_2_ (data not shown). In future studies, we will investigate the role of NHR-49 in metformin-mediated protection against mitis group streptococci.

Metformin’s protective effects can be understood within a broader framework linking conserved longevity pathways to host defense, integrating multiple metabolic and stress-response mechanisms identified in *C. elegans*. It mimics dietary restriction-like states and activates energy stress responses primarily through AMPK and LKB1, engaging downstream regulators such as SKN-1/Nrf2, NHR-49, and MDT-15 to reprogram oxidative metabolism, enhance antioxidant defenses, and promote cellular resilience [20, 40]. These effects are reinforced by lysosomal signaling including PEN2-dependent AMPK activation and induction of autophagy, as well as modulation of proteostasis via unfolded protein responses. In parallel, metformin suppresses growth-promoting pathways through inhibition of mTORC1, consistent with an evolutionarily conserved mechanism linking nutrient sensing to growth control and longevity [64]. Metformin also reshapes one‑carbon metabolism by restricting S‑adenosylmethionine and altering microbial folate and methionine pathways, linking host epigenetic regulation such as H3K4me3 modifiers to lifespan and healthspan [65, 66]. Concurrently, lipid metabolic programs such as ether lipid biosynthesis further support pro-longevity states [67]. Importantly, these metabolic and stress adaptations intersect with immunity: metformin activates the conserved PMK-1/p38 MAPK pathway and SKN-1-dependent oxidative stress responses, coupling redox homeostasis with innate immune function [25]. Together, mitochondrial stress, lysosomal signaling, mTORC1 inhibition, and p38 MAPK form an integrated, conserved network that coordinates lifespan extension with pathogen defense. Thus, metformin acts not through a single pathway but as a systems‑level modulator that synchronizes metabolism, proteostasis, epigenetic state, and immunity to promote organismal health. Given the increasing antimicrobial resistance observed in viridans streptococci, including vancomycin tolerance and daptomycin resistance, host-directed immunomodulatory strategies like metformin therapy could provide significant clinical benefits. In immunocompromised patients, such as cancer patients prone to viridians streptococcal shock syndrome, co-administration of metformin may help mitigate infection risk and severity. Future studies using mammalian models are warranted to evaluate whether metformin elicits similar SKN‑1/Nrf2-mediated protective responses via p38 MAPK and Mediator subunits.

## Conclusion

In summary, we show that metformin protects *C. elegans* against mitis group streptococci by activating an SKN-1-dependent oxidative stress response. This protective effect requires MDT-15 for full transcriptional activation of SKN-1 target genes and is associated with p38 MAPK signaling. Moreover, our study positions metformin not only as an aging and metabolic modulator but also as a potent mediator of host defenses, acting through an evolutionarily conserved pathway involving p38/pMK‑1, SKN‑1/Nrf2, and MDT‑15/MED15.

## Supplementary Information

Below is the link to the electronic supplementary material.

Supplementary Fig. 1. GO analysis of proteins identified in *mdt-15* mutant worms under metformin treatment compared with N2 worms. (A) Heatmap showing the expression patterns of proteins identified by LC–MS/MS in WT and *mdt-15* mutant worms. (B) GO biological process terms enriched in *mdt-15* mutant worms under metformin-treated and untreated conditions compared with N2 worms (PNG 437 KB)

High Resolution Image (TIF 15.2 KB)

Supplementary Table 1. List of *C. elegans* strains used in this study (DOCX 15.2 KB)

Supplementary Table 2. Comprehensive list of proteins identified by LC–MS/MS in N2 and *mdt-15* mutant worms under metformin-treated and untreated conditions (XLSX 3.72 MB).

Supplementary Table 3. GO biological process analysis of upregulated and downregulated proteins identified in N2 and *mdt-15* mutant worms under metformin-treated and untreated conditions (XLSX 52.5 KB)

Supplementary Table 4. KEGG pathway analysis of differentially regulated proteins in N2 and *mdt-15* mutant worms under metformin-treated and untreated conditions, including associated proteins for each enriched term and pathway (XLSX 32.0 KB)

## Data Availability

Most data supporting the findings of this study are available within the article. Additional data supporting the findings are available from the corresponding author upon reasonable request.
