# Supplementary material for: p38 MAPK and MDT-15 mediate SKN-1-dependent metformin-induced protection against mitis group streptococci in Caenorhabditis elegans
Source: GeroScience. 2026 Jul 27;48(6):7505–22. doi: 10.1007/s11357-026-02405-6 (PMC13638828; doi:10.1007/s11357-026-02405-6)
Supplement: Supplementary file 1 [file 11357_2026_2405_MOESM2_ESM.docx]

**Table S1 List of *C. elegans* strains used in this study**

| **Strain** | **Genotype** | **Transgene** | **Source** |
| --- | --- | --- | --- |
| N2 | *C. elegans* wild isolate |  | CGC |
| EU1 | *skn-1*(zu67) IV/nT1 [unc-?(n754) let-?] (IV;V) |  | CGC |
| AU3 | *nsy-1*(ag3) II |  | CGC |
| KU4 | *sek-1*(km4) X |  | CGC |
| KU25 | *pmk-1*(km25) IV |  | CGC |
| VC289 | *prdx-2*(gk169) II |  | CGC |
| XA7702 | *mdt-15*(tm2182) III |  | CGC |
| CL2166 | dvIs19 III | (pAF15)*gst-4*p::GFP::NLS] | CGC |
| LD002 | IdIs1 | *skn-1b/c*::GFP + *rol-6*(su1006) | Keith Blackwell |
